# A New System for Profiling Drug-Induced Calcium Signal Perturbation in Human Embryonic Stem Cell–Derived Cardiomyocytes

**DOI:** 10.1177/1087057114557232

**Published:** 2015-03

**Authors:** Kimberley J. Lewis, Nicole C. Silvester, Steven Barberini-Jammaers, Sammy A. Mason, Sarah A. Marsh, Magdalena Lipka, Christopher H. George

**Affiliations:** 1 Wales Heart Research Institute & Institute of Molecular and Experimental Medicine, School of Medicine, Cardiff University, Cardiff Wales, UK

**Keywords:** calcium signaling, cell imaging, human, cardiac, stem cells, drug discovery

## Abstract

The emergence of human stem cell–derived cardiomyocyte (hSCCM)–based assays in the cardiovascular (CV) drug discovery sphere requires the development of improved systems for interrogating the rich information that these cell models have the potential to yield. We developed a new analytical framework termed SALVO (synchronization, amplitude, length, and variability of oscillation) to profile the amplitude and temporal patterning of intra- and intercellular calcium signals in hSCCM. SALVO quantified drug-induced perturbations in the calcium signaling “fingerprint” in spontaneously contractile hSCCM. Multiparametric SALVO outputs were integrated into a single index of in vitro cytotoxicity that confirmed the rank order of perturbation as astemizole > thioridazine > cisapride > flecainide > valdecoxib > sotalol > nadolol ≈ control. This rank order of drug-induced Ca^2+^ signal disruption is in close agreement with the known arrhythmogenic liabilities of these compounds in humans. Validation of the system using a second set of compounds and hierarchical cluster analysis demonstrated the utility of SALVO to discriminate drugs based on their mechanisms of action. We discuss the utility of this new mechanistically agnostic system for the evaluation of in vitro drug cytotoxicity in hSCCM syncytia and the potential placement of SALVO in the early stage drug screening framework.

## Introduction

Cardiovascular (CV) drug development urgently needs enhanced screening systems that enable improved early stage assessments of drug hazard and more accurately predict in vivo drug toxicity and clinical efficacy.^1,2^ Multicellular syncytia of human stem cell–derived cardiomyocytes (hSCCMs, which encompass both embryonic stem cell–derived CMs [ESCMs] and induced pluripotent stem cell–derived CMs [iPSC-CMs]) have emerged as important new tools for drug screening.^1,3–5^ These models re-create, to some extent, the cell-to-cell communication that modulates cellular behavior in vivo and obviate some of the issues associated with nonhuman cell-based assays. However, there are acknowledged issues regarding their hybrid embryonic-adult proteomic and transcriptomic signatures, cellular heterogeneity postdifferentiation, intracellular microarchitecture and signaling organization, electrophysiological profiles, and pharmacological sensitivities.

The incorporation of hSCCM-based assays into the drug discovery toolkit now warrants the development of improved systems that can better interrogate the rich information that these new cellular models have the potential to yield. Moreover, the shift toward phenotypic profiling strategies, which evaluate drug bioactivities in the context of a deep understanding of cellular signaling networks, requires methodological innovation beyond the transposition of conventional optical and electrophysiological technologies onto these new cell systems.^1,2^

The calcium (Ca^2+^) signaling network underpins virtually every biological phenomenon,^6^ and thus understanding the spatiotemporal organization of Ca^2+^ signals in functionally coupled hSCCMs, under normal and drug-exposed conditions, could potentially reveal the mechanistic bases of drug-evoked phenotypic modulation and cellular (dys)function.^7–9^ Previous studies in single mammalian cells showed that subtle changes in Ca^2+^ signaling dynamics, which did not perturb steady-state Ca^2+^ homeostasis, had profound consequences for cell phenotype.^6,10–12^ In this study, we extend these approaches to profile Ca^2+^ signal organization in spontaneously contractile, functionally coupled hSCCMs. We describe the development and proof-of-concept validation of a novel system (termed SALVO: synchronization, amplitude, length, and variability of oscillations) that enables a detailed interrogation of the spatiotemporal patterning of Ca^2+^ signals in hSCCMs and the exploration of the mechanisms of drug action. We discuss the potential utility of this system in the drug safety screening landscape and how, in combination with other contemporary screening approaches, SALVO may enable a better understanding of the mechanisms underlying CV drug cytotoxicity and lead to the improved prediction of drug hazard in humans.

## Materials and Methods

### Cytiva Culture and Maintenance

Cytiva hSCCMs were derived from monolayer differentiation of an H7 hESC cell line that had been expanded under feeder-free conditions and then subjected to a proprietary differentiation protocol (GE Healthcare, Piscataway, NJ, USA).^13^ Cytiva were supplied as a heterogeneous cell population containing 50.5% ± 5.4% cardiomyocytes (*n* = 6 batches; batch numbers 4799455, 4903456, 4638600, 4636900, 7396634, 6265575). Cells were thawed and seeded into 7-mm^2^ chambers created by the adherence of silicon gaskets (CultureWell MultiWell 3 mm in diameter, 1-mm-depth inserts [Life Technologies]) on glass-bottomed culture chambers (In Vitro Scientific, Sunnyvale, CA, USA) that had been precoated with Matrigel (BD Biosciences, Franklin Lakes, NJ, USA), Franklin Lakes, NJ, USA diluted 1:30 (v/v) in Knockout DMEM (Life Technologies, Carlsbad, CA, USA). Cells were seeded as per the manufacturer’s instructions at a density of 2500 cells per mm^2^ surface area (i.e., 17,500 cells in each 7-mm^2^ chamber corrected for plating efficiency) in antibiotic-free RPMI 1640 (15 µL) supplemented with B27 (1:50 [v/v] dilution) (RPMI/B27) (Life Technologies) and maintained at 37 °C in a humidified 5% CO_2_ environment. Cells were allowed to adhere to the coverslip for 2 h before the dish was filled with RPMI/B27 (2 mL). Medium was exchanged every 48 h.

### Immunofluorescent Detection of Troponin-T

Cells were fixed (4% [v/v] formaldehyde in phosphate-buffered saline [PBS], containing [in mM] NaCl [140], KCl [2.7], Na_2_HPO_4_ [10], NaH_2_PO_4_ [2], pH 7.4) for 10 min at room temperature (RT) and then washed three times with PBS prior to permeabilization (0.1% [v/v] Triton X-100 in PBS, 4 min at RT). Nonspecific antibody interactions were blocked by incubation in horse serum (4% [v/v] in PBS, 1 h, RT) before cells were incubated with mouse anti–troponin-T (TnT; 1:200 [v/v] in PBS) (MA5-12960; Thermo Scientific, Waltham, MA, USA) overnight at 4 °C. Cells were washed with PBS (3 × 5 min) before being incubated with Alexa Fluor 546 anti–mouse IgG (1:200 [v/v]; Life Technologies) for 1 h at RT in the dark. Following washing with PBS (3 × 5 min), cell nuclei were counterstained with DAPI (1 µg/mL; 20 min) prior to further washing in PBS (2 × 1 min) and mounting under Prolong Gold (Life Technologies). Cells were imaged using a confocal microscope (SP5; Leica Microsystems, Wetzlar, Germany), and assessments of TnT positivity and cellular alignment were made using image analysis (LAS-AF [Leica Microsystems] and ImageJ [National Institutes of Health, Bethesda, MD, USA]).

### Ca^2+^ Imaging and Analysis of Ca^2+^ Signals

Cells were incubated with fluo-4 AM (5 µM) (Life Technologies) for 1 h at 37°C before coverslips were flooded with RPMI/B27 (2 mL). Fluo-4 Ca^2+^-dependent signals were visualized in 0.021-mm^2^ regions with a 63× oil immersion objective (NA 1.4) using argon laser excitation (488 nm) and a confocal microscope (SP5; Leica Microsystems). Cells were maintained at 37 °C throughout experiments. Images were recorded every 100 ms at a 512 × 512–pixel resolution. The application of caffeine (5 mM final concentration) was used to trigger sarcoplasmic reticulum (SR) Ca^2+^ release and thereby estimate the intra-SR Ca^2+^ store load.

Taking our lead from Uhlen’s method of using spectral analysis to investigate the organization of Ca^2+^ signals,^14^ we developed SALVO to decode the spatiotemporal patterning of Ca^2+^ oscillations within individual cells and across multicellular populations. SALVO outputs 30 parameters that describe Ca^2+^ signal organization,^15^ but for the purposes of this study, we focused on five parameters: oscillation rate (rate, Hz) and four other parameters that quantify the amplitude and temporal patterning of Ca^2+^ oscillation: (1) amplitude heterogeneity index (AHI) and (2) temporal heterogeneity index (THI), statistical assessments of signal amplitude and temporal variability within single cells, respectively; (3) intertransient noise (ITN), defined as the Ca^2+^ signal variability occurring *between* Ca^2+^ oscillations and that extends the use of signal variability (SV) to measure point-by-point differences in very low-amplitude Ca^2+^ signals^6,10,12^; and (4) synchronization, an index of the temporal coincidence of Ca^2+^ oscillation maxima occurring across cells in the population. The calculation of these parameters is described in **Supplementary Figure S1**. SALVO is implemented using a Python-based computer program.^15^ The detection of signal maxima and minima over a 30-s period in data obtained from 6 to 20 cells in each instance was performed using SALVO’s autodetection algorithms (based on spline detect or threshold detection methods) or by manual assignation of the start, peak, and end of each Ca^2+^ spike. SALVO outputs were archived and interrogated using a custom-built SQL-based database system (DB Miner, CHG, NCS, SBJ).

### Characterization of Drug-Evoked Ca^2+^ Perturbation

Baseline assessments of Ca^2+^ signals in fluo-4–loaded cells prior to the addition of drug (control) were established as described above. Cells were then sequentially exposed to increasing concentrations of cardioactive drugs, selected to represent those categories assigned by Redfern and colleagues^16^ as category 1 (“repolarization-prolonging as an intended, desirable effect”; sotalol, prescribed as a β-blocker with class III antiarrhythmic properties^17^), category 2 (“drugs that have been withdrawn or suspended from the market in at least one major regulatory territory due to an unacceptable risk of TdP”; astemizole and cisapride, QT-prolonging IK_r_/hERG-blocking antihistamine and gastric prokinetic, respectively), category 3 (“drugs that have a measurable incidence of TdP in humans”; thioridazine, a drug with the highest incidence of TdP among prescribed antipsychotics via off-target effects on IK_r_/hERG,^18^ and flecainide, a torsadogenic class IC antiarrhythmic [Na_v_1.5 channel blocker]^17^), and category 5 (“no published reports of TdP in humans”; nadolol, a nonselective β-blocker). Valdecoxib, a COX-2–selective nonsteroidal anti-inflammatory drug (NSAID) that was initially approved for use in the treatment of osteoarthritis and rheumatoid arthritis but was subsequently withdrawn from the market due to adverse CV effects,^19^ was not included in the assessment by Redfern et al.^16^ but was included here. We purchased rofecoxib (Vioxx) from Sequoia Research Products (Pangborne, UK), but the supplied compound exhibited inconsistent bioactivity in our assay system.

Stock solutions of drugs (10 mM) in ultrapure water or tissue-culture grade DMSO were diluted in RPMI/B27, and the concentration of each drug under test was sequentially increased. After each addition and following a period of equilibration (approximately 90 s), fluo-4–dependent Ca^2+^ signals were acquired for 30 s. Using this protocol, the same population of cells was imaged throughout the entire drug addition sequence.

To validate the system, we used a second set of compounds at maximal effective concentrations in our assay (1–30 µM): category 1, amiodarone (class III antiarrhythmic); category 2, terodiline (QT-prolonging proarrhythmic); category 5, metoprolol (selective β_1_-blocker); celecoxib (COX-2 inhibitor), and aconitine (non–QT-prolonging proarrhythmic). We also included two drugs that have mechanisms of actions distinct from those above: verapamil (L-type Ca^2+^ channel blocker, category 5) and ranolazine (late I_Na_ current inhibitor). In all experiments, operators were blinded to the identities of the drugs under test throughout all phases of postexperimental data analysis.

### Electrophysiological Recordings of Action Potentials in Cytiva

Cells in 35-mm glass-bottomed culture dishes containing a superfusion insert (AutoMate PCP-1; Digitimer Ltd., Welwyn Garden City, UK) were mounted on the stage of an inverted microscope (CKX-41; Olympus, Tokyo, Japan) and were superfused with normal Tyrode (NT) solution (containing [in mM] NaCl [145], KCl [4], MgCl_2_ [1], CaCl_2_ [2], HEPES [10], glucose [10]; pH 7.4 with NaOH) or NT-containing drugs maintained at 37 °C using a heated jacket.

Cells were whole-cell clamped with 3- to 4-MΩ patches and an intrapipette solution (containing in [mM] KCl [120], MgCl_2_ [1.75], CaCl_2_ [5.37], EGTA [10], HEPES [10], and Na_2_ATP [4]; pH 7.2 with KOH) in current-clamp (IC) mode using a CV-7B headstage and MultiClamp 700B amplifier controlled by MultiClamp software (Molecular Devices, Sunnyvale, CA). Data were digitized and acquired using a Digidata 1322a card and pClamp software, respectively (Molecular Devices). Action potentials were sampled at a rate of 20 kHz and low pass (Bessel) filtered at 10 kHz.

Spontaneous cell contracture was terminated using blebbistatin (5 µM, 10 min), an agent that inhibits myofilament shortening and thereby negates patch-clamp recording instability but preserves normal Ca^2+^ cycling.^20^ Following cessation of contractures, spontaneous action potentials were observed in all cells following the application of a current pulse to obtain whole-cell access. Action potential (AP) recordings from blebbistatin-immobilized cells were taken under control (no-drug) conditions (1 min) and following the switch to drug-containing NT. APD_90_, corrected for AP cycle length using Fridericia’s method (APD_90(corr)_ = APD_90_/cycle length^0.3^), was calculated from five consecutive APs following a 3-min exposure of the cells to drug.

### Hierarchical Clustering and Statistical Analysis

Hierarchical clustering of SALVO outputs was performed using the uncentered correlation similarity metric and centroid clustering method in Cluster 3.0 (Lawrence Berkley Laboratories, CA, USA) with output visualization using TreeView (v1.1.6r4; JAM Software, Trier, Germany). Data sets were tested for normality using the D’Agostino-Pearson algorithm, and normally distributed data were compared using analysis of variance with intergroup comparisons performed using Bonferroni’s post hoc test. Nonnormally distributed data were compared using the Kruskal-Wallis test with Dunn’s post hoc test. For comparing only two groups of data, two-tailed Student *t* test (normal) or the Mann-Whitney test (nonnormal) was used. All statistical analysis was performed using Prism 6.0 (GraphPad Software, La Jolla, CA, USA). All data are given as mean ± standard error (SE). A *p* value of 0.05 was considered statistically significant.

## Results and Discussion

### Profiling the Functional Maturation of Ca^2+^ Handling

**Figure 1.**
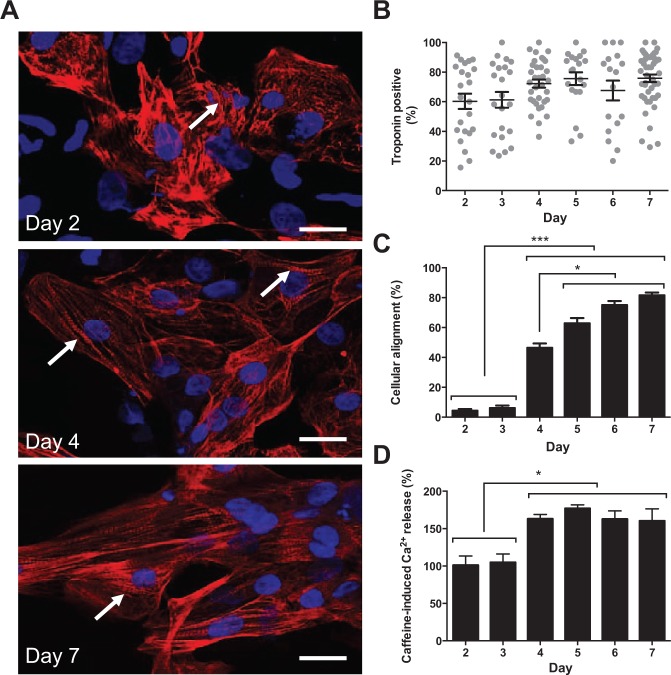
Phenotypic characterization of Cytiva. (**A**) The subcellular distribution of troponin-T (TnT; red) was visualized. Cell nuclei were stained with DAPI (blue). Arrow indicates TnT striation. Scale bar = 20 µm. (**B**) The proportion of TnT-positive cardiomyocytes (*n* = 28–40 separate image fields). (**C**) The relative alignment of cells. The maximum value (100%) represents every cell aligning along a single axis. Data are mean ± SE (*n* = 23–48 image fields). *p < 0.05. ***p < 0.001. (**D**) The amplitude of Ca^2+^ release triggered by caffeine (5 mM). Data are mean ± SE (*n* > 4 fields of view, *n* > 12 cells in each instance). *p < 0.05.

The relative axial alignment of TnT-positive cardiomyocytes (CMs) was low on days 2 and 3 postseeding but progressively increased to a maximum at day 5 with no further change to day 7 (**Fig. 1A–C**). This alignment of cells was associated with the intracellular redistribution of TnT that transitioned from a form that lacked apparent organization (day 2) into well-defined striatal arrangements (days 4–7) (**Fig. 1A****,****C**). The proportion of TnT-positive CMs remained constant between days 2 and 7, suggesting a stable population of differentiated CMs and the absence of proliferative or differentiation-competent contaminant non-CM cells (**Fig. 1B**). Cells between days 2 and 7 exhibited robust responses to caffeine, and contrary to a report that only a subset of hSCCMs is caffeine sensitive (approximately 38%),^21^ all Cytiva CMs possessed the requisite cellular machinery to support caffeine-induced Ca^2+^ release even by day 2 postseeding (**Suppl. Fig. S2**). However, the amplitude of caffeine-induced Ca^2+^ release, an index of the functional Ca^2+^ storage capacity of the SR, was augmented at day 4 and remained unchanged through day 7 (**Fig. 1D** and **Suppl. Fig. S2**). CMs between days 4 and 7 also exhibited a faster rate of Ca^2+^ sequestration/extrusion after caffeine-induced Ca^2+^ release (**Suppl. Fig. S2**).

Our data point to the culture-dependent alignment of CMs, the intracellular reorganization of TnT, and the improved functional capacity of the Ca^2+^ handling machinery and corroborate reports of progressive SR maturation in cultured hSCCMs.^21^ Although our study did not establish the causal drivers of these phenomena, we observed that day 4 CMs exhibited maximal amplitude and kinetics of caffeine-induced Ca^2+^ release (**Fig. 1D** and **Suppl. Fig. S2**), yet were incompletely aligned (**Fig. 1C**). This suggests a complex association between changes in cellular morphology, subcellular architecture, and functional modulation of the SR Ca^2+^ store that requires further investigation. The progressive alignment of cells from day 3 was not associated with increased levels of cell death in the population that remained consistently low (cell death [%]: day 3, 2.0 ± 0.8; day 4, 1.1 ± 0.4; day 5, 1.5 ± 0.4; day 6, 1.4 ± 0.6; day 7, 1.9 ± 0.5; *p* = 0.8573).

**Figure 2.**
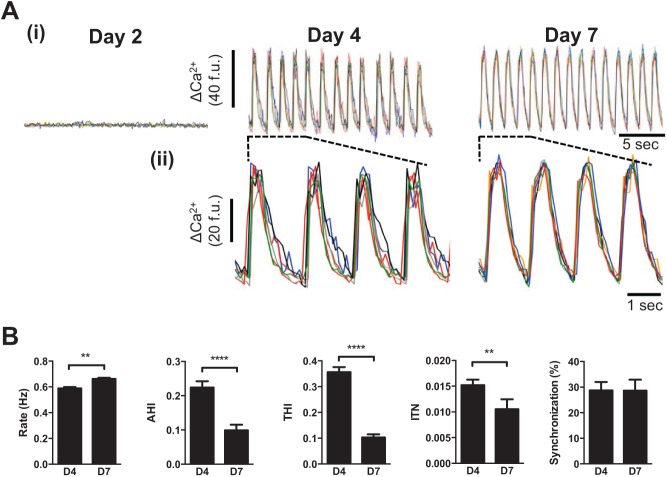
Functional maturation of Ca^2+^ cycling in Cytiva. (**A**) (**i**) Ca^2+^ traces from six representative cells. (**ii**) Expanded sections depicted by dashed lines in (**i**). Data for days 2 through 7 are given in **Supplementary Figure S3**. (**B**) A five-parameter description of Ca^2+^ signal organization in spontaneously oscillating cells. Data are from ≥5 separate experiments. **p < 0.01. ****p < 0.001. Data from days 4 through 7 are given in **Supplementary Figure S3**.

The morphological and Ca^2+^ signaling changes observed at day 4 coincided with the onset of spontaneous Ca^2+^ oscillations (53.6% ± 19.7% cells on day 4 exhibited spontaneous Ca^2+^ oscillations vs. 0% on day 3, *p* < 0.0001) and synchronized contraction (**Fig. 2A(i)** and **Suppl. Movies 1–3**). Between days 4 and 7, the proportion of cells exhibiting spontaneous Ca^2+^ release remained static (day 5, 65.0% ± 18.9%; day 6, 64.8% ± 14.3%; day 7, 59.2% ± 13.1%; *p* = 0.505), but the Ca^2+^ oscillations became qualitatively (**Fig. 2A(ii)**) and quantitatively (**Fig. 2B**) better organized (see also **Suppl. Fig. S3**). Notably, there was a marked decrease in AHI and THI (indices that tend to zero with reduced amplitude and temporal variability of spontaneous Ca^2+^ release) and a reduction in ITN, indicative of less variability in low-amplitude Ca^2+^ signals occurring between large Ca^2+^ transients (**Fig. 2B**). Taken together, the attenuation of AHI, THI, and ITN suggests an improvement in the amplitude and temporal organization of Ca^2+^ oscillations between days 4 and 7. These data extend our findings that pivotal changes in cell morphology, alignment, and Ca^2+^ signaling that occur between days 3 and 4 drive the onset of spontaneous Ca^2+^ oscillations, which become progressively better organized to day 7. Intercellular synchronization of Ca^2+^ oscillations remained unchanged between days 4 and 7 (**Fig. 2B**), indicating that functional cell-to-cell coupling established by day 4 was unaffected by subsequent phenotypic changes. Cell density, which varied between 200 and 1000 cells/mm^2^ depending on batch number and the plating efficiency of the cells, had no measureable effect on the Ca^2+^ handling behavior of the cells or on the extent of intercellular synchronization (**Suppl. Fig. S4**). It has been reported that hSCCMs plated at “low density” (500–1200 cells/mm^2^) exhibit markers of cellular hypertrophy and electrical remodeling.^22^ Although our mean cell densities were below this threshold, the alignment of cells in culture leads to regional heterogeneity, with some regions at very high densities and other areas that contain few cells (see **Fig. 1**). Since there was no increase in cell death to day 7, the contribution of cell death to the observed distribution of cells can be excluded. We did not investigate the functional impact of more uniformly distributed regions of high cell densities (>1200 cells/mm^2^) on Cytiva CM phenotype.

The optimal functional state of Cytiva CMs (i.e., like that determined on days 6 and 7; **Suppl. Fig. S3**) existed only for a short period. Beyond day 7, the proportion of TnT-positive cells progressively decreased, and by day 14, there was a pronounced functional deterioration in Ca^2+^ signaling (**Suppl. Fig. S5**). At day 21, the few cells that remained adherent (typically 5%–10% of those present at day 7) had mostly reverted to fibroblastic-like morphologies with a loss of TnT striation and organization (**Suppl. Fig. S5**). This short window meant that we performed all subsequent experiments on CMs between days 6 and 8. We did not pursue strategies reported to prolong hSCCM functionality (e.g., optimized replating cycles^23^), and it is likely that such protocols could improve the functional longevity of Cytiva in culture.

### Using Ca^2+^ Signal Perturbation to Assess Drug-Induced Cytotoxicity

**Figure 3.**
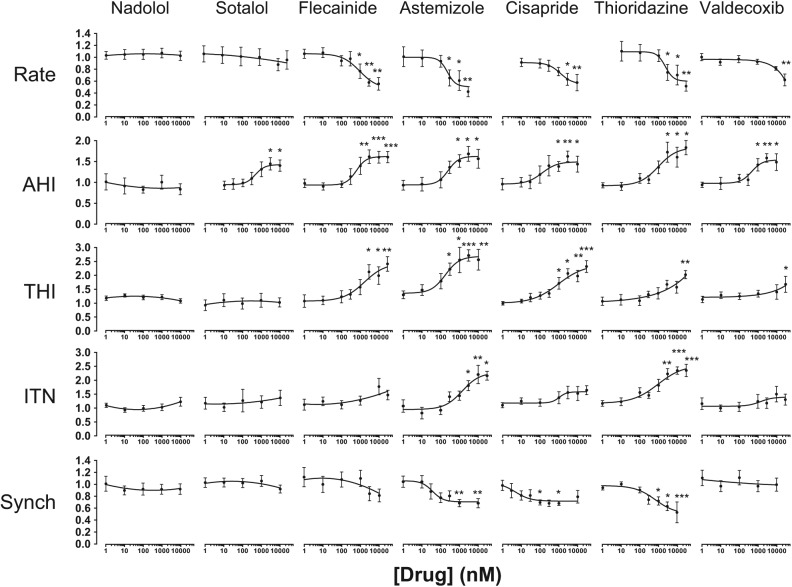
Profiling cardiovascular drug-evoked perturbations in Ca^2+^ signals. Spontaneously oscillating cells were exposed to compounds (1 nM–30 µM) (see Materials and Methods). Data were normalized to controls (no drug) and are plotted as mean ± SE (*n* ≥ 6). *p < 0.05. **p < 0.01. ***p < 0.001. EC_20_ values, the concentration of drug that produces a 20% change relative to control values, are given in **Supplementary Table S2**.

The disruption of the spatiotemporal patterning of Ca^2+^ signals is a hallmark of cellular dysfunction, and so we next investigated the propensity of cardioactive drugs to perturb intra- and intercellular Ca^2+^ signal organization. Nadolol, a nonselective β-blocker with an excellent clinical safety profile, had no measurable effect on Ca^2+^ handling at concentrations up to 30 µM (**Fig. 3** and **Suppl. Table S2**). Sotalol, a class III antiarrhythmic that has been reported to have QT-prolonging effects at therapeutic plasma concentrations exceeding 10 µM, caused an isolated change in AHI at concentrations >1 µM but did not reduce the extent of intercellular synchronization (**Fig. 3** and **Suppl. Table S2**). Concentration-response profiling revealed that drugs with recognized proarrhythmic hazards resulting from hERG-blocking activities (astemizole, cisapride, thioridazine) or via other “non-hERG” mechanisms (flecainide, valdecoxib) evoked large effect sizes in multiple parameters of Ca^2+^ handling (**Fig. 3** and **Suppl. Table S2**). These data confirmed that the perturbation of cellular Ca^2+^ signals in vitro is a hallmark feature of drugs with known cardiovascular risk in humans irrespective of whether their proarrhythmic mechanism of action involves IK_r_/hERG blockade. Notably, SALVO quantified the Ca^2+^-disrupting effects of valdecoxib, a drug that had hitherto not been considered to exert direct effects on CMs using conventional electrophysiological assessments. Our data are consistent with those of Pyrko and colleagues,^24^ who reported the elevation of cytoplasmic Ca^2+^ within seconds of the addition of 2,5-dimethyl-celecoxib, an analogue valdecoxib, to glioblastoma cells. These findings add weight to the concern that contemporary electrophysiological measurements do not adequately resolve potential CV risk associated with some drugs, especially those that do not directly modulate cellular Na^+^ and K^+^ ion handling. Illustrating this point, impedance measurements in hSCCM failed to identify the potential hazard associated with rofecoxib (Vioxx),^25^ an analogue of valdecoxib that was withdrawn because of unacceptable CV risk.^19^ We believe that SALVO has the potential to occupy this void in the contemporary drug screening landscape.

**Figure 4.**
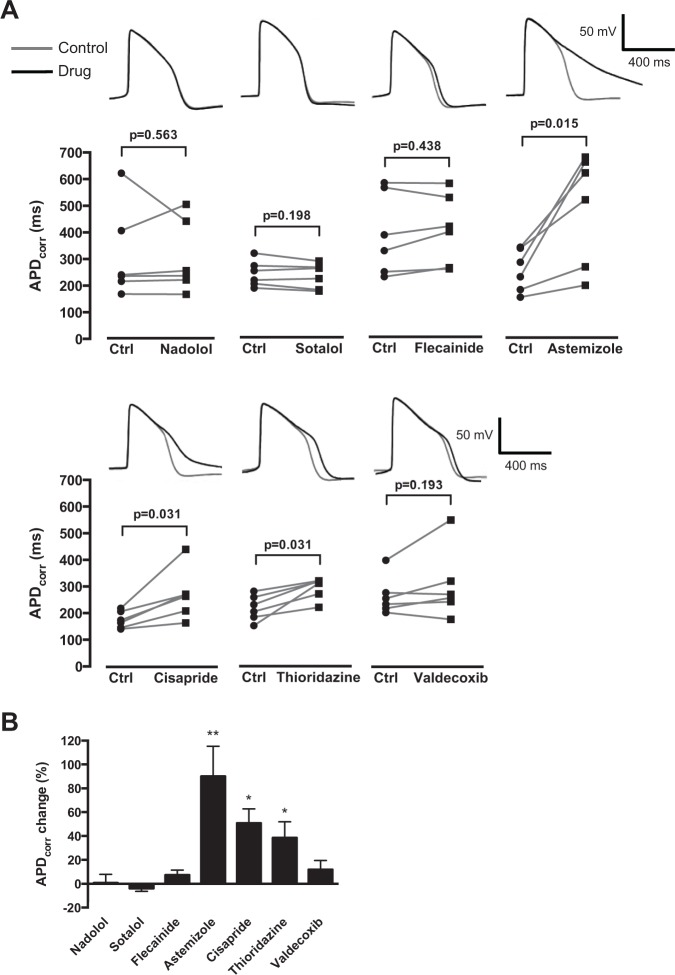
Investigating the effect of cardiovascular-active drugs on Cytiva APD_90_. (**A**) Cells were exposed to drugs at EC_20_ concentrations for 3 min (**Suppl. Table S2**). APD_90_ values were corrected for beat period using Fridericia’s algorithm. Data points represent individual paired experiments (*n* = 6). Representative control (gray) and drug-treated (black) action potential traces are shown. (**B**) APD_90_ plotted as the percentage change relative to the paired control value (no drug). *p < 0.05. **p < 0.01.

To further corroborate the utility of SALVO to identify potential CV drug hazard that arises from diverse mechanistic bases, we profiled the effects of these same drugs on spontaneous APs. Cytiva CMs were characterized by resting membrane potentials of approximately −60 mV and exhibited ventricular-like waveforms (**Fig. 4**). Astemizole, cisapride, and thioridazine increased APD90, entirely in keeping with their known IK_r_/hERG blocking activities. However, flecainide and valdecoxib, drugs that markedly perturbed Ca^2+^ signals (**Fig. 3**) and are known to increase proarrhythmic susceptibility in some circumstances,^16,19^ had no effect on action potential duration (**Fig. 4A****,****B**). These data reinforce the use of SALVO to profile compounds based on their disruption of cellular Ca^2+^ signal organization irrespective of whether the drugs in question modulate the electrophysiological profile of hSCCMs.

Cytiva are supplied as a heterogeneous mix of CMs and non-CMs. It is therefore plausible that effects of drugs on contaminant non-CMs could influence the measured effect on CMs (e.g., EC_20_ values). We used a fluorescence-activated cell sorting (FACS)–based strategy to enrich CMs, but the small population of viable yet morphologically abnormal CMs that were obtained offset any improvement in Ca^2+^ signal organization observed in post-FACS CMs (**Suppl. Fig. S6**).

### Calculation of a SALVO Toxicity Score and Its Alignment with Proarrhythmic Risk in Humans

**Figure 5.**
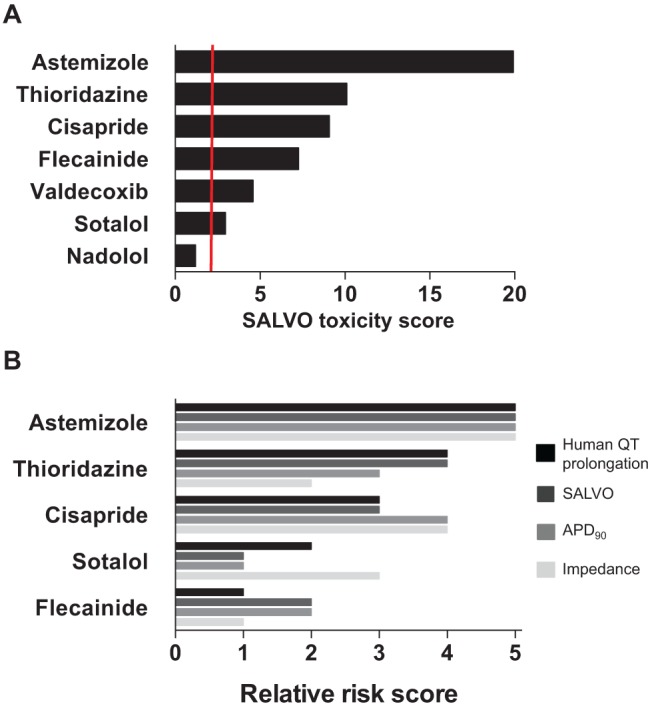
Integrating multiparametric outputs into the SALVO toxicity score. (**A**) The calculation of the SALVO toxicity score is described in **Supplementary Figure S7**. The vertical red line indicates a score twice that of control (no-drug) values. (**B**) A rank-ordered comparison of three in vitro assessments of cardioactive drugs (SALVO, APD_90_, and impedance measurements) against established in vivo drug QT prolongation liability (human QT prolongation). SALVO and APD_90_ data are from the present study, impedance measurements are from Guo et al.,^25^ and QT prolongation is from Redfern et al.^16^ Nadolol and valdecoxib were not profiled in the Guo or Redfern studies and are not included here.

The five-parameter SALVO output (**Fig. 3**) was integrated into a single in vitro cytotoxicity score (SALVO toxicity score). The rank order of the extent of Ca^2+^ perturbation (astemizole > thioridazine > cisapride > flecainide > valdecoxib > sotalol > nadolol) was in very close agreement with the known proarrhythmogenic liabilities of these drugs in humans^13,16,25,26^ (**Fig. 5A** and **Suppl. Fig. S7**). Moreover, SALVO exhibited a superior correlation with established CV drug risk in humans compared with electrophysiological readouts (APD_90_, impedance measurements). Thus, for the reasons outlined above, we propose that SALVO will improve early stage assessments in CV drug hazard liabilities. However, the extrapolation of in vitro cell-based toxicity outputs to risk prediction in clinical scenarios is problematic,^1,2^ and it is accepted that, at present, no single approach can accurately translate in vitro readouts to drug effects in humans. Consequently, SALVO outputs should be appropriately integrated with information obtained from other platforms (e.g., electrophysiology).

There is also the issue regarding the relevance of drug potency determined in vitro to clinical dosing in humans. Guo and colleagues^25,27^ used an impedance-derived index of arrhythmic beating in hSCCMs to derive a predicted proarrhythmic score (PPS) that took into account the therapeutically relevant total concentration of drug in human plasma (defined as C_eff_). More recently, Clements and Thomas^13^ used a similar approach to generate a predictive risk score (PRS) based on their measurement of field potential duration (FPD) in Cytiva. In keeping with these studies, we adjusted the five-parameter SALVO output to account for the free plasma concentration of the clinically effective drug (ETPC_unbound_) (**Suppl. Tables S1** and **S2**). Our resultant PPS (ETPC_unbound_/EC_20_) (**Suppl. Fig. S8**) described a rank order of thioridazine > cisapride > valdecoxib that was entirely consistent with the SALVO toxicity scores (**Fig. 5A**) and aligned closely to the recognized hazards that these drugs pose in humans. However, flecainide exhibited an elevated PPS at odds with its relatively low proarrhythmia risk in humans. Moreover, anomalous PPS values for sotalol (PPS ~15) and astemizole (PPS <1) grossly misrepresent the CV hazard associated with sotalol (low) and astemizole (high)^16^ (**Suppl. Fig. S8**). The erroneous values for these two drugs, which were also noted by Clements and Thomas,^13^ result from skew introduced by very high and low ETPC_unbound_ values (14,733 and 0.2 nM for sotalol and astemizole, respectively) (**Suppl. Table S1**). Consequently, our data highlight the significant potential for the misleading categorization of “risk” following the calculation of a PPS or PRS (i.e., the adjustment of in vitro toxicity scores using clinically relevant plasma drug concentrations). These data strongly suggest that such simple indices are unlikely to accurately predict in vivo risk/hazard. However, by studying a larger set of compounds with known risk, together with consideration of other factors that influence in vivo drug responses, our future understanding of the relationship between in vitro assay outputs and CV risk in humans should be improved.

### Hierarchical Clustering Analysis of SALVO Outputs to Discriminate Mechanisms of Drug Action

**Figure 6.**
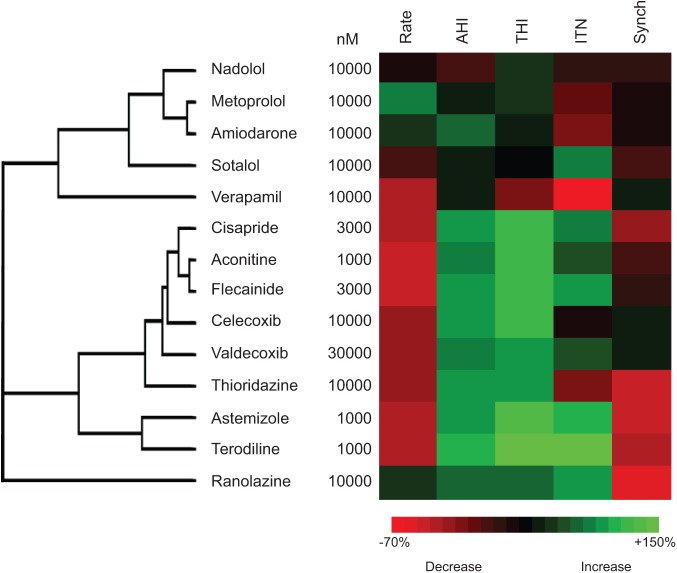
Hierarchical cluster analysis of compounds based on their mechanism of action. The effects of drugs, including a secondary set of validation compounds (see Materials and Methods), on Ca^2+^ cycling parameters were determined at their maximal effective concentrations.

To further validate the system and to investigate the ability of SALVO to discriminate drugs based on their mechanisms of action, we tested a second set of compounds (see Materials and Methods). Hierarchical cluster analysis (HCA) revealed that, as anticipated, drugs with comparable mechanisms of action clustered together—for example, hERG blockers (terodiline with astemizole), non-hERG blockers (aconitine with flecainide), COX-2 inhibition (celecoxib with valdecoxib), class III antiarrhythmics (sotalol with amiodarone), and β-blockers (nadolol with metoprolol) (**Fig. 6**). Importantly, we studied two cardioactive drugs selected on the basis of distinct mechanisms of action to those compounds used above. Verapamil, a clinically safe L-type Ca^2+^ channel blocker, reduced the rate of Ca^2+^ oscillation at very high concentrations of drug (>10 µM) but otherwise had no measurable effect on cellular Ca^2+^ handling. Ranolazine, an inhibitor of the late I_Na_ that is not associated with elevated CV hazard,^28^ reduced intercellular synchronization against a background of unchanged Ca^2+^ cycling. Consistent with their different mechanisms of action, verapamil and ranolazine were separated in the resultant dendrogram and did not cluster with the other compounds tested in this study (**Fig. 6**). These data support the conclusion that SALVO discriminates compounds based on their mechanism of action. This analysis used data obtained from the maximally effective concentration of drug to reconcile the impact of these compounds on cellular Ca^2+^ signaling with their known mechanisms of action. However, these concentrations (typically >1 µM; see **Fig. 6**) greatly exceed the free plasma levels of drug achieved via clinical dosing regimes.^16,29^ We emphasize therefore that **Figure 6** shows the clustering of drugs based on their mechanisms of action and not on their established safety/risk profile in humans.

### Future Directions

This study reports the development and proof-of-concept validation of SALVO, a new system to quantify drug-induced (dys)organization of intra- and intercellular Ca^2+^ signaling in hSCCM populations. We propose that SALVO fills a current void in the early stage drug screening process, specifically via its ability to identify those compounds that exhibit CM cytotoxicity (and thus potentially increase proarrhythmic susceptibility) but have negligible impact on the cellular electrophysiological profile. We also demonstrate the utility of SALVO to discriminate drugs based on their mechanism of action. The present work used only a small number of compounds of known proarrhythmia risk, and clearly, a systematic evaluation of a much larger panel of compounds is now warranted.

The comparative assay miniaturization reported here—17,500 cells seeded in 7-mm^2^ gaskets and with the potential to use fewer cells still—suggests that this system is amenable to scaling to higher throughput formats (e.g., 384-well plates). Moreover, the system could be configured toward “multiplexing” ion-sensitive fluorescent probes or in combination with electrophysiological platforms.^30^ Outside of the predictive toxicology arena, such detailed assessments of cellular Ca^2+^ signaling may also be useful in quality control (QC) processes, cellular phenotyping, and profiling batch-to-batch variability during the manufacture/differentiation process. It is also noteworthy that SALVO analysis could be applied to *any* oscillatory biological signal. To this end, it is of interest to explore SALVO in the context of other cell systems (e.g., hepatocytes) and to the analysis of other cellular signals that change on different timescales (e.g., pH).

## Supplementary Material

Supplementary material

## Supplementary Material

Supplementary material

## Supplementary Material

Supplementary material

## Supplementary Material

Supplementary material
