# Supplementary material for: A New System for Profiling Drug-Induced Calcium Signal Perturbation in Human Embryonic Stem Cell–Derived Cardiomyocytes
Source: J Biomol Screen. 2015 Mar;20(3):330–40. doi: 10.1177/1087057114557232 (PMC4361473; doi:10.1177/1087057114557232)
Supplement: Supplementary material [file Supplementary_Information.pdf]

Supplementary Information

"A new system for profiling drug-induced calcium signal perturbation in human embryonic stem cell-derived cardiomyocytes".

Kimberley J. Lewis, Nicole C. Silvester, Steven Barberini-Jammaers, Sammy A. Mason, Sarah A. Marsh, Magdalena Lipka, Christopher H. George

Supplementary Tables

Supplementary Table 1 : Calculating the free plasma concentration of cardioactive drugs
Supplementary Table 2 : EC_{20} values for Ca^{2+} perturbation

Supplementary Figures

Supplementary Figure 1: Profiling Ca^{2+} signals using SALVO
Supplementary Figure 2: The duration of caffeine-evoked Ca^{2+} release in Cytiva
Supplementary Figure 3: Functional maturation of Ca^{2+} handling in Cytiva
Supplementary Figure 4: The organization of Ca^{2+} signals is independent of cell density
Supplementary Figure 5: Deterioration of Ca^{2+} handling beyond Day 7
Supplementary Figure 6: FACS enrichment of CMs
Supplementary Figure 7: Calculation of the SALVO toxicity score
Supplementary Figure 8: Calculation of the predicted proarrhythmic score (PPS)

Supplementary Movies

All movies are compiled from 30 seconds of image data at 20 frames per second (i.e. 2x speed).

Supplementary Movie 1: Day 3 post-seeding (Batch 7396634)
Supplementary Movie 2: Day 4 post-seeding (Batch 7396634)
Supplementary Movie 3: Day 7 post-seeding (Batch 7396634)

Supplementary Table 1. Calculating the free plasma concentration of cardioactive drugs.

The maximal effective therapeutic plasma concentration of each drug (ETPC) described by Redfern et al. (Redfern et al. (2003) Cardiovasc. Res. 58:32-45) was used to calculate the levels of free drug ($ETPC_{unbound}$). ETPC for nadolol and valdecoxib were from Krukemyer et al. (Pharm. Res. (1990) 9:953-956) and Yuan et al. (Drug Metab. Disp. (2002) 30:1013-1021), respectively. The values for drug-protein binding were obtained from DrugBank (www.drugbank.ca).

**Drug**	**ETPC (nM)**	**Free drug (%)**	**$ETPC_{unbound}$ (nM)**
Nadolol	387	70	271
Sotalol	14733	100	14733
Flecainide	1931	60	1159
Astemizole	8	2	0.2
Cisapride	129	2	3
Thioridazine	1781	5	89
Valdecoxib	2257	2	45

Supplementary Table 2. EC_{20} values for Ca^{2+} perturbation.

Drug concentrations (nM) that produced a 20% change from control values (no drug) were calculated for each SALVO parameter from regression analysis of the data shown in Figure 3. The effective therapeutic plasma concentration of free drug ($ETPC_{unbound}$, nM) was calculated in Supplementary Table 1.

	Rate	AHI	THI	ITN	Synch
**Nadolol**	> 30000	> 30000	> 30000	> 30000	> 30000
**Sotalol**	> 30000	210	> 30000	> 30000	> 30000
**Flecainide**	215	260	285	2110	> 30000
**Astemizole**	50	100	55	320	15
**Cisapride**	445	40	105	>30000	5
**Thioridazine**	1120	205	530	110	120
**Valdecoxib**	9870	685	12800	>30000	> 30000

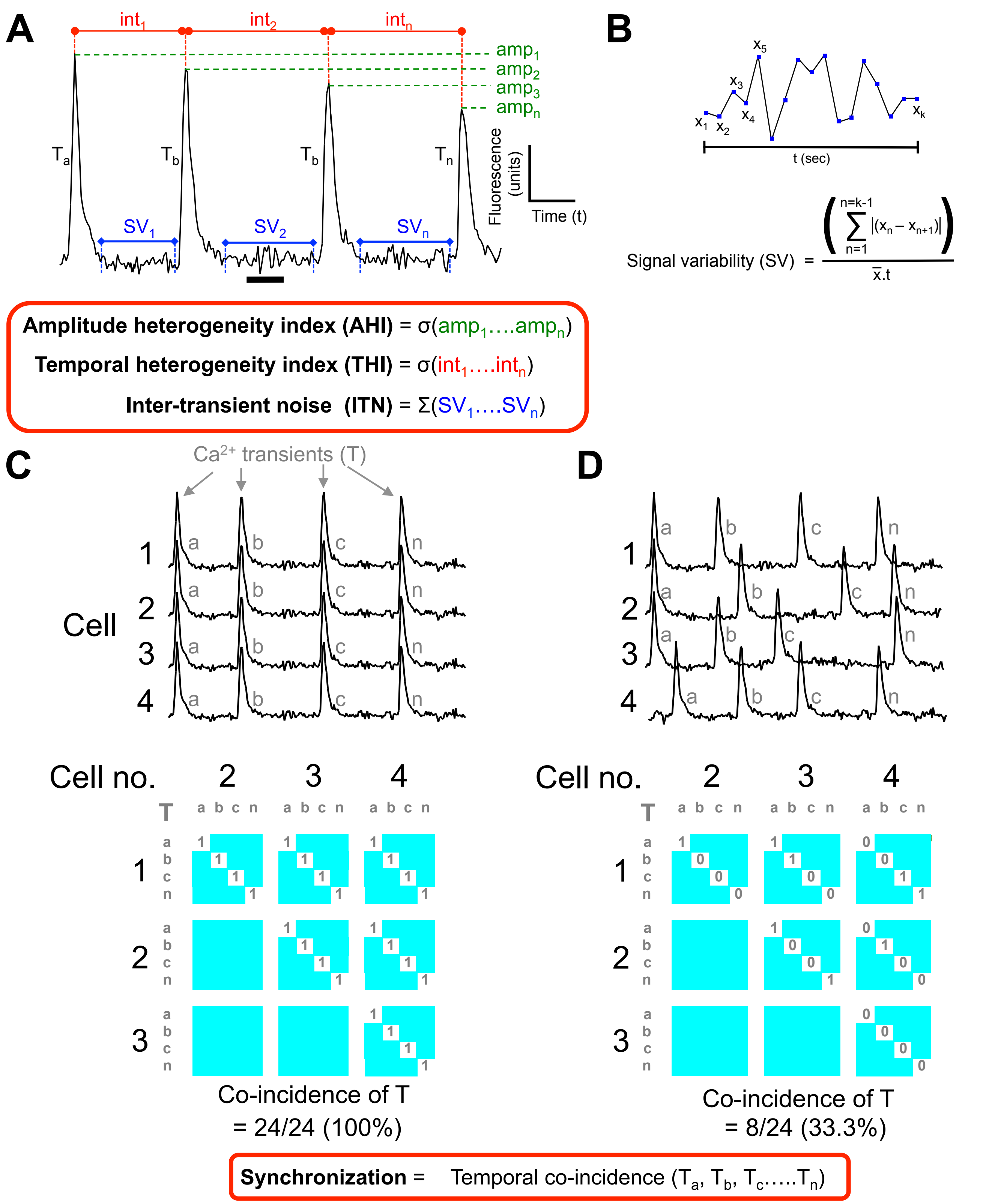

Supplementary Figure 1. Profiling Ca^{2+} signals using SALVO.
(A) A schematic description of the methods for calculating AHI, THI and ITN based on the analysis of amplitude and temporal characteristics of Ca^{2+} oscillations. AHI and THI will tend to zero with decreasing variability in Ca^{2+} oscillation amplitude and temporal regimentation. **(B)** Signal variability (SV), a core component of ITN, is calculated from the point-by-point differences in a Ca^{2+} signal (George et al. (2006) Circ. Res. *98*:88-97; George et al. (2007) Circ. Res. *100*:874-883). **(C)** and **(D)** Intercellular synchronization of Ca^{2+} signal maxima (transients, T) was calculated using a simple matrix system. In **(C)** all four oscillations (a,b,c,n) are temporally co-incident across the four cells (#1-4) and thus synchronization is maximal (24/24 events, 100%). In **(D)**, the co-incidence of T across cells #1-4 is reduced to 8/24 events (33.3%).

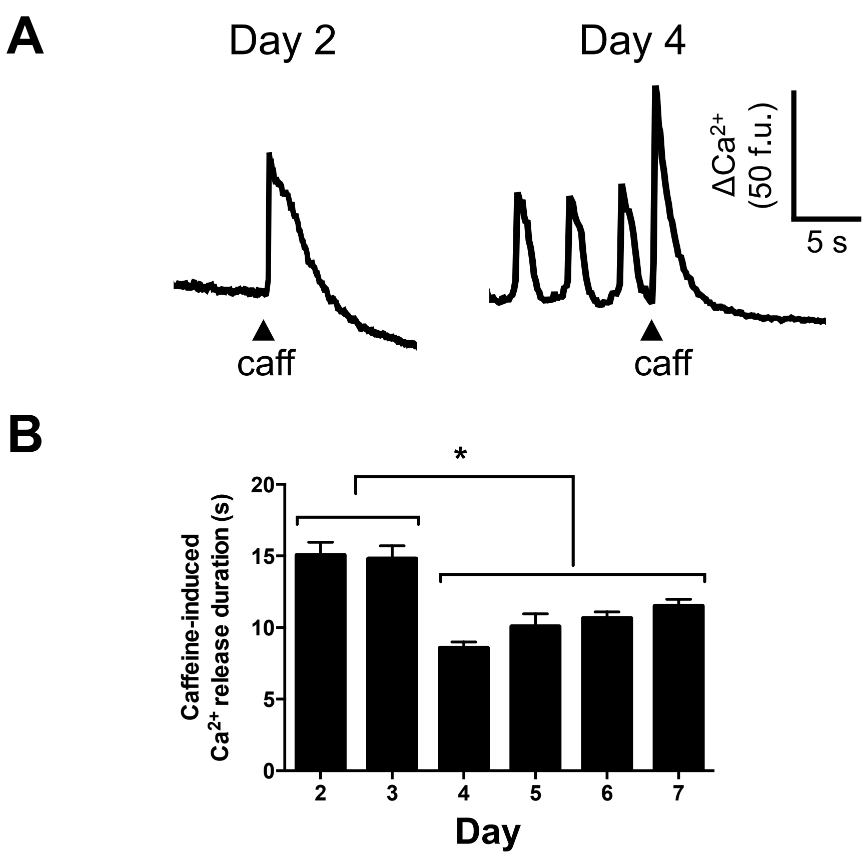

Supplementary Figure 2. The duration of caffeine-evoked Ca^{2+} release in Cytiva.
(A) Caffeine (5 mM, arrowhead) was used to trigger Ca^{2+} release in non-oscillatory Cytiva (Days 2 and 3) and in Cytiva supporting robust spontaneous Ca^{2+} oscillations (Days 4 to 7). **(B)** The duration of caffeine-triggered Ca^{2+} release was calculated and is given as mean ± SE (n > 4 experiments, > 12 cells per experiment). The amplitude characteristics of caffeine-induced Ca^{2+} release is given in Figure 1D. * $p < 0.05$.

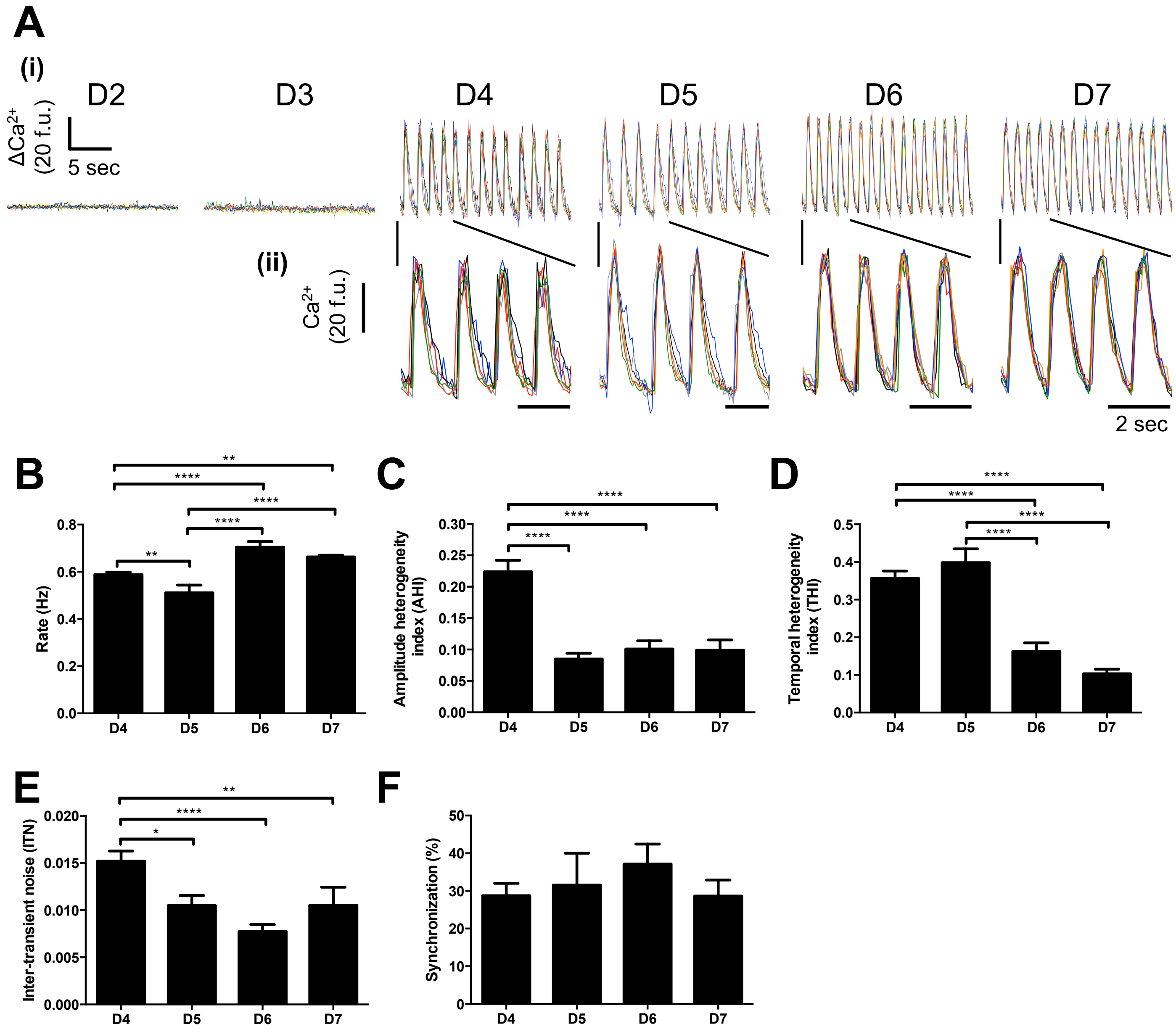

Supplementary Figure 3. Functional maturation of Ca^{2+} handling in Cytiva.
(A) (i) Ca^{2+} traces from six representative cells on Days 2 to 7 in culture. ***(ii)*** Expanded sections of traces from ***(i)***. ***(B – F)*** A five-parameter description of the temporal organization of intra- and intercellular Ca^{2+} handling in spontaneously oscillating cells between Days 4 and 7 (n ≥ 5 separate experiments). * $p < 0.05$, ** $p < 0.01$, and **** $p < 0.001$.

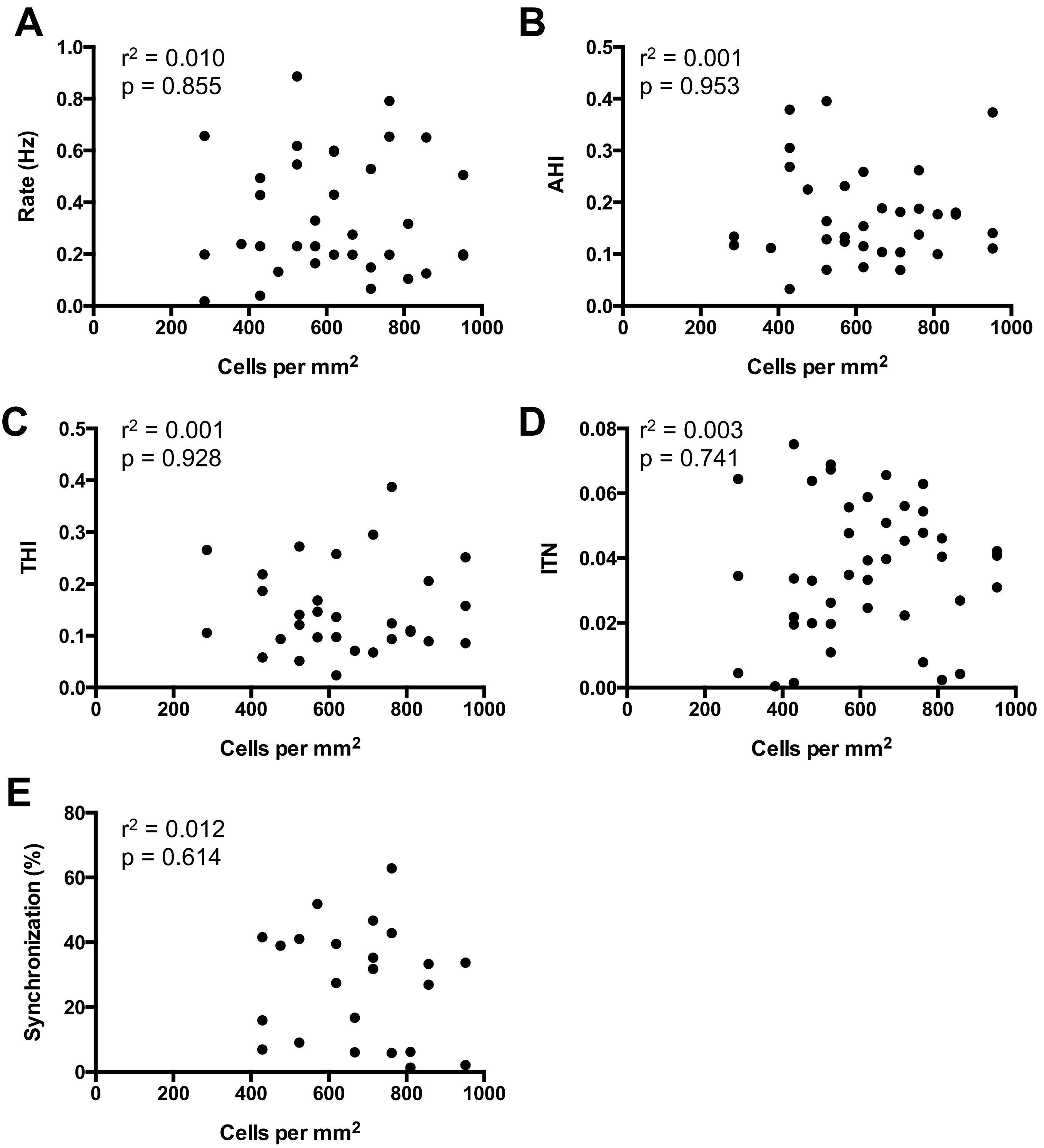

Supplementary Figure 4. The organization of Ca^{2+} signals is independent of cell density.

(A - E) Scatter plots of SALVO outputs against cellular density at Day 7. Linear regression coefficients (r^2) and the probability of the slope being significantly non-zero (p) were calculated. Each data points represents a separate cell seeding.

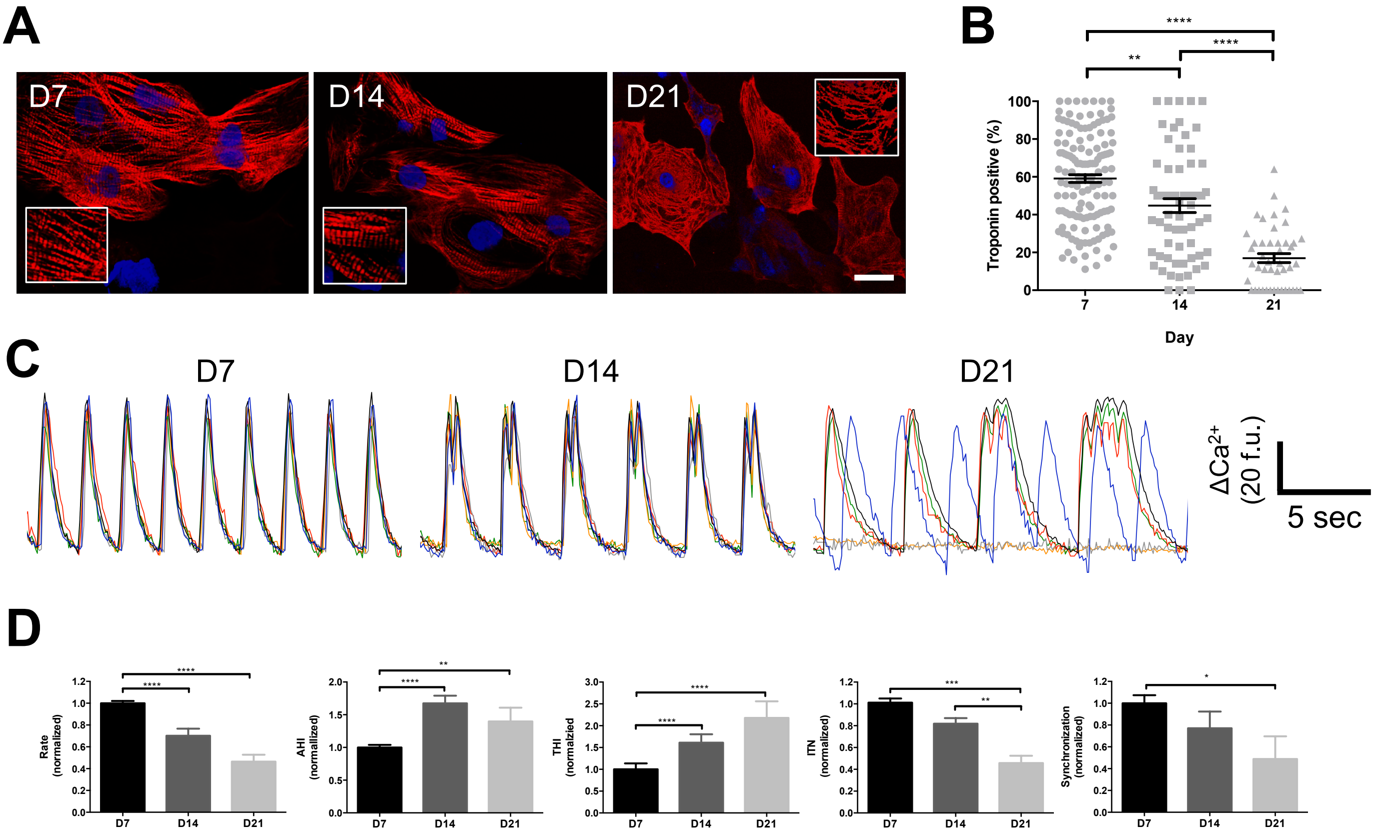

Supplementary Figure 5. Deterioration of Ca^{2+} handling beyond day 7.
(A) Immunofluorescent staining of TnT organization (red) in Day 7, 14 and 21 cells. Cell nuclei were stained with DAPI (blue). White boxes depict the organization of TnT in more detail. Scale bar = 20µm. **(B)** The proportion of TnT-positive cells on Days 7, 14 and 21. Each data point represents the analysis of separate image fields. ** $p < 0.01$ and ****$p < 0.0001$. **(C)** Qualitative deterioration in the amplitude and temporal organization of Ca^{2+} oscillations in six representative cells at Days 14 and 21 compared to Day 7. **(D)** Quantitative SALVO-based analysis of Ca^{2+} handling on Days 7, 14 and 21. Data were normalized to Day 7 values (assigned 1) in each instance (n ≥ 6 separate experiments).
*$p < 0.05$, ** $p < 0.01$, *** $p < 0.001$ and **** $p < 0.0001$.

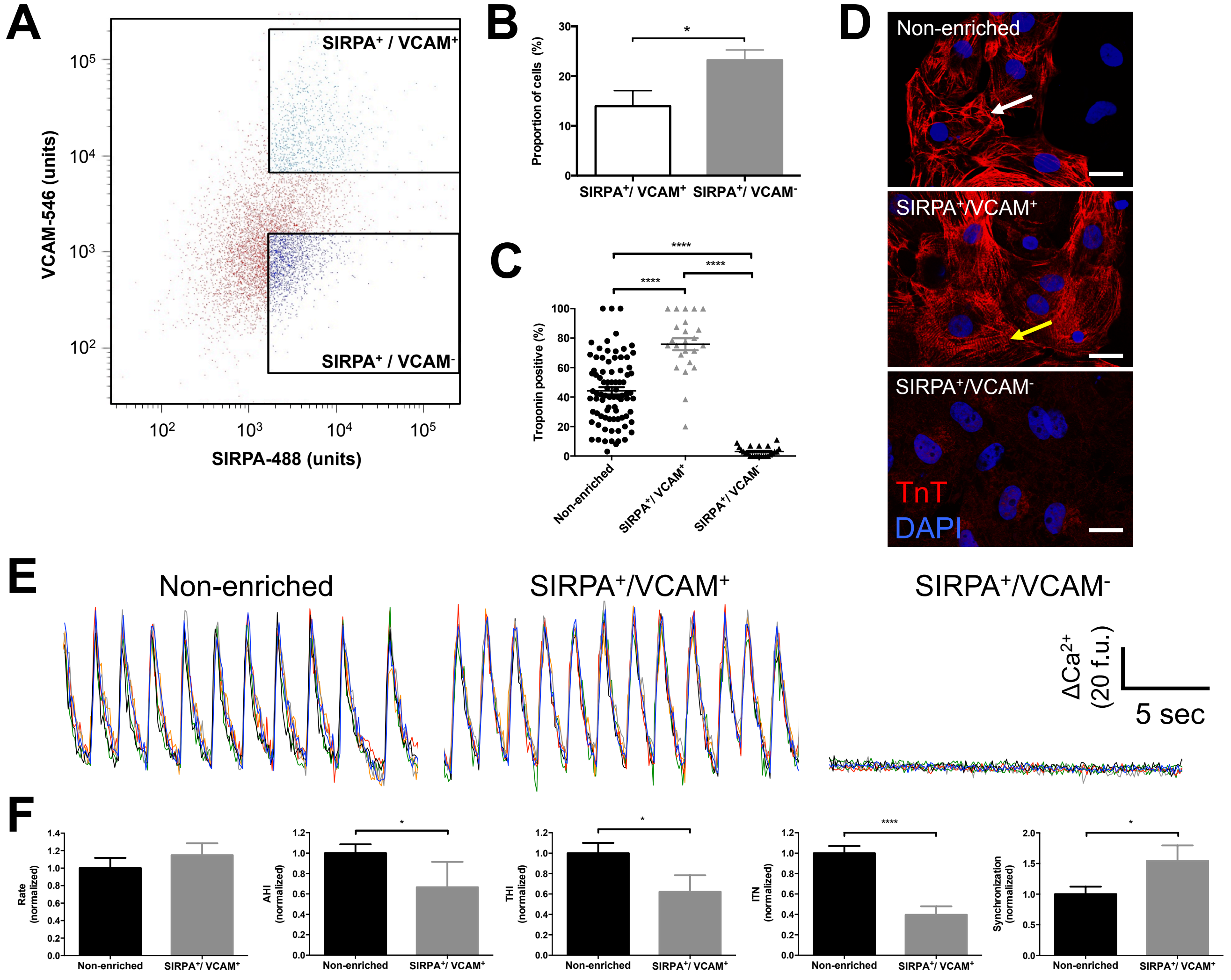

Supplementary Figure 6. FACS enrichment of CMs using SIRPA and VCAM immunolabelling. **Methods:** Immediately after thawing, cells were pelleted (400 x g; 4mins) and re-suspended with biotin-labelled anti-CD172a (signal regulatory protein alpha (SIRPA) (Dubois et al. (2011) Nat. Biotechnol. 29:1011-1018)) (1:500 (v/v) in PBS) (MRC OX-41 Thermo Scientific) plus/minus mouse anti-VCAM1 (1:200 (v/v) in PBS) (MA1-91815 Thermo Scientific) for 30min with continual agitation. Cells were washed and incubated with streptavidin AlexaFluor 488 conjugate (1:40 (v/v) in PBS) and/ or AlexaFluor 546 anti-mouse IgG (1:200 (v/v) in PBS) (Life Technologies) for 30mins in the dark with continued agitation. Cells were pelleted (400 x g; 4mins), rinsed in PBS and re-suspended in pre-warmed RPMI/B27 (37°C) and sorted using 488nm (SIRPA) and 543nm (VCAM) laser lines at 2000 events per second through an 85µm nozzle (BD FACSAria III). As a control for the FACS procedure, non-labelled cells were processed via FACS and were gated on the basis of viability (side-scatter) but were not actively sorted using fluorescence (‘non-enriched’). Post-FACS, cells were seeded into 7mm^2 silicon-gasketed Matrigel-coated coverslips as described in Methods.

Results: Viable SIRPA$^+$/VCAM$^+$ populations **(A)** that comprised 14 ± 3% total cell population **(B)** and characterized by increased TnT positivity (76 ± 4% TnT-positive cells compared with 44 ± 2% non-enriched controls, $p < 0.0001$) **(C)** were obtained. SIRPA$^+$/VCAM$^+$ CMs exhibited abnormal morphology, reduced axial alignment and decreased intracellular organization of TnT as consequence of the FACS process **(D)** (arrows depict the lack of TnT organization (white arrow) or the striatal arrangement of TnT (yellow arrow). Scale bar = 20µm. In addition, the FACS procedure reduced cell viability (66 ± 5% and 31 ± 7% viability on Days 2 and 7 post-FACS SIRPA$^+$/VCAM$^+$ (n = 6 sorts) versus 97 ± 1% and 96 ± 1% in non-FACS Cytiva populations, $p < 0.01$). However, SIRPA$^+$/VCAM$^+$ CMs supported robust well-organized Ca^{2+} oscillations in an increased proportion of the cells (59 ± 12% versus 35 ± 4% in non-enriched cells, 1.7 fold increase $p < 0.05$) **(E).** SALVO analysis of Ca^{2+} oscillations in Day 7 SIRPA$^+$/VCAM$^+$ CMs suggest quantifiable improvements in Ca^{2+} handling in enriched CM populations (n ≥ 5 separate experiments) **(F)**. * $p < 0.05$ and **** $p < 0.0001$.

Calculate percentage effect size **(E)** for each SALVO parameter, normalized to control conditions, at each drug concentration

↓

Effect size **(E)** is weighted according to magnitude of drug-induced change at each concentration **(λ)** to give **(E_w)**

(see Box 1)

↓

Cumulative E_w for drug concentrations of 0.1, 1, 10, 100 and 1000 nM for each SALVO parameter **(E_S)**

↓

Cumulative E_S across all SALVO parameters (**E_{drug}**)

(see Box 2)

↓

Normalization of E_{drug} to control cells reported as SALVO toxicity score

E_w = E x λ

Effect size (E,%)	λ
<5	1.00
5-10	1.10
10-20	1.21
20-30	1.33
30-40	1.46
40-50	1.61
50-60	1.77
60-70	1.95
70-80	2.14
>80	2.36

The relationship between **E** and **λ** is given by the second order polynomial equation:

$$\lambda = 1.015+0.0081E+0.00007.5E^2$$

Box 1

E_{drug}= E_S(Rate) + E_S(AHI) + E_S(THI) + E_S(ITN) + E_S(Synchronization)

Box 2

Supplementary Figure 7. ***Calculation of the SALVO toxicity score.***
The weighting factor (λ) was arbitrarily increased by 10% for every 10% increase in effect size 'bin' (> 10% effect size) (Box 1).

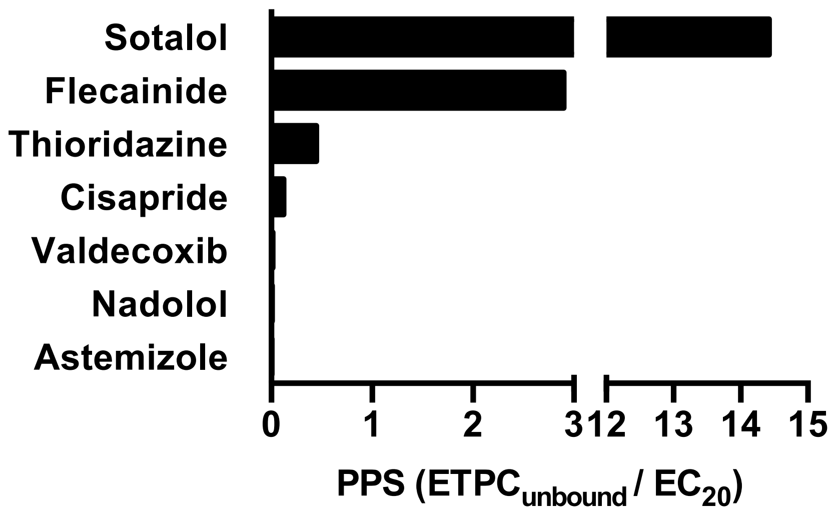

Supplementary Figure 8. Calculation of the predicted proarrhythmic score (PPS).
The PPS (ETPC$_{unbound}$ / EC$_{20}$) was calculated from the values given in Table 1 as described by Guo and colleagues (Guo et al. (2011) Toxicol. Sci. *123:*281-289).
